# Measuring cognitive engagement in physical education: scale development and validation

**DOI:** 10.3389/fpsyg.2026.1874125

**Published:** 2026-07-15

**Authors:** Meng Zhang, Yu Deng, Nan Zheng, Kaiyuan Wang, Ji Wu

**Affiliations:** 1 School of Physical Education, Shanghai University of Sport, Shanghai, China; 2 School of Economics and Management, Shanghai University of Sport, Shanghai, China

**Keywords:** cognitive engagement, ICAP framework, middle school students, physical education, scale development

## Abstract

**Background:**

Cognitive engagement in physical education is an important factor that reflects the quality of physical education teaching and influences students' learning outcomes. Effectively assessing students' cognitive engagement in physical education is valuable for classroom evaluation and instructional intervention. However, existing instruments for measuring cognitive engagement in physical education remain limited in contextual specificity, measurement accuracy, and practical applicability. Based on the ICAP framework, this study aimed to develop and validate the Cognitive Engagement in Physical Education Scale (CEPE) for Chinese middle school students.

**Methods:**

The scale was initially developed based on a literature review and classroom observations. A full reliability and validity test was conducted with 855 middle school students in Jinan, China.

**Results:**

The final CEPE consisted of 14 items across four dimensions: observation and execution, cognitive processing, self-regulation, and collaborative innovation. The four-factor model showed acceptable fit to the data: χ^2^/ df = 4.396, RMSEA = 0.080, GFI = 0.919, CFI = 0.952, TLI = 0.938, IFI = 0.952, and SRMR = 0.041. Standardized factor loadings ranged from 0.68 to 0.97. The composite reliability values were all above 0.80, and the average variance extracted values were all above 0.50, supporting convergent validity. Although the Fornell-Larcker criterion was not fully satisfied for cognitive processing and self-regulation, additional evidence from HTMT values and competing model comparisons supported the discriminant validity of the four-factor structure. The CEPE total score and subscale scores were positively correlated with physical education learning self-efficacy, providing evidence of criterion-related validity.

**Conclusion:**

The CEPE developed in this study based on the ICAP framework showed acceptable reliability and validity. It may provide a useful measurement tool for assessing students' cognitive engagement in physical education.

## 1 Introduction

Cognitive engagement in physical education (PE) refers to the mental effort and learning strategies that students actively use to understand physical education and health knowledge and to master motor skills during physical education classes (Greene, 2015; Wang et al., 2019). On the one hand, cognitive engagement in physical education is viewed as a key indicator of students' learning quality and teachers' instructional quality (Skinner et al., 2008). On the other hand, because it is highly malleable (Fredricks et al., 2004), it is also regarded as an important means of achieving instructional goals and improving teaching effectiveness in physical education (Hastie et al., 2022). More importantly, compared with other types of student engagement, cognitive engagement in physical education, as a substantive form of engagement, can predict students' learning achievement more effectively and accurately (Hastie et al., 2022; Newmann, 1992). Recent studies further suggest that cognitive engagement in physical education predicts students' learning outcomes mainly through a direct pathway in which students complete deeper cognitive tasks, such as relating and reasoning (Nystrand and Gamoran, 1991; Wang et al., 2019). Basic cognitive participation, by contrast, plays an indirect role by supporting these deeper processes. This highlights the importance of guiding students to engage in high-quality cognitive processing tasks and improving their cognitive engagement in physical education. With the development of physical literacy and the growing emphasis on higher-order thinking (Brown et al., 2020), cognitive engagement in physical education has received increasing attention from policymakers, school administrators, physical education teachers, and other stakeholders in school physical education (Zhu et al., 2009; Noetel et al., 2023). Researchers and practitioners in school physical education are actively promoting innovation in physical education classes. They aim to improve students' cognitive engagement in physical education by changing teaching methods and using digital and intelligent technologies to support instruction (Cui et al., 2024). One strategy to promote innovation related to cognitive engagement in physical education is to provide educators with tools to measure their success in facilitating student cognitive engagement in physical education.

Scientifically sound measurement tools can help assess students' cognitive engagement in physical education and evaluate the effectiveness of interventions. They can also expand the research field of cognitive engagement in physical education and promote further development of related studies. However, current tools for measuring cognitive engagement in physical education have clear limitations. First, some studies have inferred students' cognitive engagement from their performance on physical education knowledge tests (Wang et al., 2019). Although this approach is relatively targeted, it is time-consuming, requires considerable expertise from test administrators, and is therefore difficult to apply in large-scale assessments. Second, most existing measures of cognitive engagement in physical education are adapted from scales originally developed for academic subjects (Leo et al., 2023). Although these adapted scales often show acceptable reliability and validity, they may not fully capture the specific features of physical education learning and may therefore measure students' cognitive engagement in PE less accurately. Third, existing scales do not classify cognitive engagement into different levels. Therefore, when researchers use these scales to measure students' cognitive engagement in physical education, they can only judge the level of engagement based on total scores (Cui et al., 2024). This makes it difficult to identify students' specific levels of cognitive engagement accurately. More importantly, existing measures have not fully captured the embodied and situated nature of cognitive engagement in physical education. Unlike many academic subjects, learning in physical education occurs through motor activity and sensory perception, teacher-student and peer interaction. Students' cognitive engagement is often expressed through PE-specific behaviors, such as observing demonstrations, mentally simulating movements, adjusting practice strategies, reflecting on movement errors, and co-constructing tactics with classmates. General cognitive engagement scales may not adequately represent these behaviors. Therefore, a PE-specific instrument is needed to assess not only the overall level of students' cognitive engagement but also the different forms and depths of cognitive engagement that occur in PE classes.

Recently, the ICAP framework has attracted increasing attention as a theory of cognitive engagement (Vosniadou et al., 2023; Özbek et al., 2024). This framework links students' modes of participation in learning activities with their cognitive processes and learning outcomes (Chi and Wylie, 2014). It provides a model for distinguishing different levels of cognitive engagement (Xu and Qiu, 2025). Specifically, the ICAP framework classifies students' cognitive engagement into four levels based on their observable behaviors in learning contexts. From lower to higher levels, these are passive engagement, active engagement, constructive engagement, and interactive engagement (Chi et al., 2018). The ICAP framework also provides clear operational criteria for each dimension. For example, in the passive mode of cognitive engagement, students only receive information from learning materials passively and do not perform any observable learning-related actions. These levels are especially relevant to physical education because students' cognitive engagement in PE is often reflected in observable learning behaviors, such as observing demonstrations, imitating movements, mentally rehearsing techniques or tactics, monitoring performance, reflecting on errors, and interacting with peers. Therefore, the ICAP framework can help distinguish different depths of cognitive engagement in PE classes and provide clear criteria for item development.

To address these gaps, the present study aimed to develop and validate the Cognitive Engagement in Physical Education Scale (CEPE) for Chinese middle school students based on the ICAP framework. The CEPE differs from existing cognitive engagement scales in three important ways. First, it is grounded in the ICAP framework and therefore conceptualizes cognitive engagement as a hierarchical construct ranging from lower-level observation and execution to higher-level collaborative innovation. Second, it is specifically designed for physical education and includes PE-specific cognitive behaviors, such as observing movement demonstrations, mentally simulating movements and tactics, monitoring practice, reflecting on errors, and designing new tactics or movements with classmates. Third, it provides dimension-level information rather than relying only on a total score, allowing researchers and teachers to identify students' specific forms of cognitive engagement in PE classes. In this way, the CEPE may provide a more context-sensitive and theoretically grounded tool for assessing cognitive engagement in physical education.

## 2 Participants

This study aimed to develop an assessment tool for measuring cognitive engagement in physical education among Chinese middle school students. To this end, a combination of purposive and cluster sampling was used to ensure the relevance and contextual accuracy of the scale content. Data were collected in two independent waves from 907 middle school students in two schools in Jinan, China, between May and July 2025. These students were selected because their schools ranked among the top in the city in physical fitness assessment results and had high-quality physical education and health courses. This sampling strategy was useful for the initial development of the CEPE because these schools provided relatively well-structured PE classes in which different levels of cognitive engagement could be observed. However, this selection may also have introduced selection bias, as students in these schools may have had higher engagement levels and more supportive instructional environments than those found in average or lower-performing schools. After invalid questionnaires were excluded, 855 valid responses remained. The first sample consisted of 341 valid responses and was used for item analysis and EFA. The second sample consisted of 514 valid responses and was used for CFA and validity testing. Of the participants, 50.5% were male (*N* = 432), 48.3% were female (*N* = 413), and 10 participants did not report their gender. Grade 7 students accounted for 59.4% of the sample (*N* = 508), Grade 8 students accounted for 31.3% (*N* = 268), and Grade 9 students accounted for 8.8% (*N* = 75). Four participants did not report their grade level.

This study was approved by the Ethics Committee of Shanghai University of Sport (102772025RT091).

## 3 Research procedure

The development of the Cognitive Engagement in Physical Education Scale closely followed DeVellis's recommendations in Scale Development: Theory and Applications (DeVellis, 2017). These recommendations were adopted because they are authoritative and are especially suitable for developing measurement tools in educational contexts. DeVellis suggested that scale development should follow eight steps. These steps include clearly defining the construct to be measured, generating an item pool, determining the measurement format, asking experts to review the items, adding validation items, selecting and administering the scale to participants, testing reliability and validity, and optimizing the length of the scale. In the following sections, this study describes the iterative process of scale development according to the steps recommended by DeVellis.

## 4 Criterion-related validity measure

The Physical Education Self-Efficacy Scale was used to measure students' confidence in learning physical education and health knowledge in physical education classes (Pintrich, 1991). In this study, the scale was scored on a five-point Likert scale. Criterion-related validity was considered acceptable if physical education self-efficacy scores were positively correlated with the scores of the Cognitive Engagement in Physical Education Scale and its dimensions.

## 5 Data analysis

Data were analyzed using SPSS 26.0 and AMOS 24.0.

First, descriptive analyses were compiled to describe the sociodemographic characteristics of the participants. Second, item analysis for the CEPE was conducted using corrected item-total correlations, following the methodological approach outlined by Wu (2010). Next, exploratory factor analysis was conducted using SPSS 26.0 to identify the factor structure of the scale. Before factor extraction, the Kaiser–Meyer–Olkin (KMO) measure of sampling adequacy and Bartlett's test of sphericity were used to examine the suitability of the data for EFA. Principal axis factoring was used as the extraction method because this study aimed to identify latent constructs underlying the CEPE items. Given that the four dimensions of cognitive engagement were theoretically expected to be correlated, direct oblimin rotation was applied, with the delta value set to 0. The pattern-matrix factor loadings were evaluated according to the criteria proposed by Comrey and Lee (2013): 0.32 poor, 0.45 fair, 0.55 good, 0.63 very good, and 0.71 excellent. Internal consistency was assessed using Cronbach's alpha coefficients. Next, confirmatory factor analysis was conducted using AMOS 24.0 to evaluate the structural validity of the scale. We adopted the model fit criteria proposed by Hu and Bentler (1999) and Sathyanarayana and Mohanasundaram (2024). Indices such as the chi-square statistic (χ^2^) and related degrees of freedom (df), comparative fit index (CFI), root mean square error of approximation (RMSEA), and standardized root mean square residual (SRMR) were used to assess the goodness of fit of the model in this step. CFA was used further to examine the factorial structure of the CEPE. Goodness-of-fit indices for an acceptable model fit were as follows: CFI ≥0.90, RMSEA ≤ 0.08, and SRMR ≤ 0.08, with a 90% confidence interval (CI). Subsequently, the constructs' convergent validity and discriminant validity were examined using a series of statistical methods. Convergent validity is used to assess whether items that are related are actually observed to be related in the data. We evaluated convergent validity using three key indicators: standardized factor loadings, average variance extracted (AVE), and composite reliability (CR). Factor loadings should exceed 0.50, indicating that the items account for a substantial portion of the variance in their respective construct (Hair, 2010). The AVE, which represents the average amount of variance a construct explains in its items, should be greater than 0.50, suggesting that the construct accounts for more than 50% of the variance in its indicators (Fornell and Larcker, 1981; Hair, 2010). The CR, which is used to assess the internal consistency of items measuring a given construct, should exceed 0.70, indicating good construct reliability (Hair, 2010). Discriminant validity is used to assess whether constructs that are theoretically distinct are also empirically distinct. We examined discriminant validity using several established methods.

## 6 Step 1: determine clearly what it is you want to measure

The first step in scale development is to think “clearly about the construct being measured” (DeVellis, 2017, p. 105). Obvious though it may sound, it is particularly important in determining the operational definition of the construct to measure and the theoretical framework to draw from (Benson and Clark, 1982). Step 1 is important in defining how the intended new instrument differs from any other existing instrument. Identifying the appropriate theoretical framework is germane to item specification and development.

In the existing literature, researchers have defined cognitive engagement in physical education from different perspectives. From the perspective of strategy use, some researchers define cognitive engagement in physical education as the process in which students use complex rather than superficial learning strategies during physical education learning (Cheon and Reeve, 2015). From the perspective of psychological states, some researchers argue that cognitive engagement in physical education refers to the degree of mental effort that students invest in physical education learning tasks (Wang et al., 2019). Other researchers suggest that students' cognitive engagement in physical education should be represented by both their attention and their use of learning strategies (Simonton and Shiver, 2021). These different definitions show that cognitive engagement in physical education is an emerging and important research area that needs further exploration. At the same time, inconsistency in definitions has led measurement tools to focus on different aspects of the construct. This makes it difficult for researchers to compare findings across studies. Therefore, it is necessary to clarify the definition and structure of cognitive engagement in physical education (Appleton et al., 2006). This can guide the whole process of scale development and help define the research scope. It can also distinguish the newly developed tool from existing instruments (Appleton et al., 2006).

Based on an extensive review of the literature and practical experience, this study defines cognitive engagement in physical education as the mental effort and learning strategies that students actively use to understand physical education and health knowledge and to master motor skills in physical education classes. It can be represented by a series of observable behavioral responses and internal thinking processes. Regarding its structure, this study drew mainly on the ICAP framework, which describes behavioral responses across four levels of cognitive engagement. Accordingly, cognitive engagement in physical education was initially divided into four levels: passive engagement, active engagement, constructive engagement, and interactive engagement. The ICAP framework was adopted because it has been widely applied to various learning activities and closely matches the characteristics of physical education learning.

## 7 Steps 2, 3, and 4: generate an item pool, determine the format for measurement, and expert review

In step 2 of DeVellis's model, the developer “generates a large pool of items that are candidates for eventual inclusion in the scale.” It is important to generate items that reflect the survey's purpose in sufficient quantity and redundancy.

This study developed the initial items through a literature review and classroom observations (Ma, 2022; Kong and Lu, 2012; Barlow et al., 2020; Pintrich and De Groot, 1990; Theodosiou, 2004). Specifically, this study first reviewed cognitive engagement instruments used in previous research. Items from instruments with good reliability and validity were then used as references. Relevant items were adapted and assigned to the corresponding dimensions of cognitive engagement in physical education according to the needs of this study. In addition, classroom observations were conducted in high-quality demonstration physical education and health classes in middle schools in Shanghai. Shanghai was selected as the site for classroom observations because it has made substantial progress in physical education curriculum reform and has developed a relatively mature and high-quality physical education curriculum system. In particular, Shanghai has promoted diversified physical education in junior middle schools and has emphasized sport skill learning, classroom practice, cooperation, and competition. These features provided rich classroom situations for observing students' cognitive engagement behaviors. Therefore, high-quality demonstration physical education classes in Shanghai were considered suitable for generating observable indicators and refining the initial item pool.

The observations focused on recording students' behavioral performance in class. Students' behaviors were classified according to the ICAP definitions of different levels of cognitive engagement. These classifications were used to generate observable indicators of students' cognitive engagement in physical education. Based on these indicators, the initial items were refined and expanded. It should be emphasized that, when assigning items to each dimension of cognitive engagement in physical education, the researchers carefully considered how the cognitive behavior verbs defined in the ICAP framework were related to observable indicators in physical education. These indicators included imitation, practicing along with the teacher, and changing the rhythm of practice. This process helped ensure the rationality of the classification and its consistency with the ICAP framework. For example, students' imitation of the teacher's demonstrated movements while listening to instruction was matched with the ICAP behavior of highlighting text. This item was used to capture students' active cognitive engagement. In addition, this study generated multiple items to capture the same construct (Clark and Watson, 2016). This approach was used to increase measurement tolerance and improve measurement accuracy. According to the principles of scale development, the initial item pool should contain three to four times as many items as the final scale (Benson and Clark, 1982). In this study, the final scale was expected to include four dimensions, with four items in each dimension. Therefore, the initial item pool needed to include at least 48 items. Based on key literature and classroom observations, this study generated an initial item pool containing 60 items. Examples of items from the initial item pool and their sources are shown in Table 1.

**Table 1:** Sample items from the initial scale item pool and their sources.

Dimension	Item	Source
Passive engagement	I watched the teacher's movement demonstration throughout the class.	Classroom observations, scale for the achievement of educational objectives in movement skills teaching
	I followed the teacher's instructions in class.	Classroom observations
	I listened to discussions among my classmates.	Classroom observations
	I listened to the teacher's explanation of the key points of the movement.	Classroom observations, physical education classroom metacognitive process questionnaire
Active engagement	I restated the key points of the movement explained by the teacher.	Classroom observations, physical education classroom metacognitive process questionnaire
	When the teacher explained and demonstrated a movement, I imitated it on my own.	Classroom observations, scale for the achievement of educational objectives in movement skills teaching
	When the teacher explained a tactic, I tried to mentally perform the tactic.	Interviews
	When the teacher explained a movement, I paid special attention to details such as the movement path and body position.	Interviews, physical education classroom metacognitive process questionnaire
Constructive engagement	I summarized the key points of the movement explained by the teacher in my own words.	Interviews, physical education classroom metacognitive process questionnaire
	I compared differences between my movements and my classmates' movements.	Interviews, physical education classroom metacognitive process questionnaire
	I set my own learning goals based on the teacher's goals and my own situation.	Self-regulated learning strategy questionnaire for middle school students, physical education classroom metacognitive process questionnaire
	I reflected on the mistakes I made during practice.	Interviews, self-regulated learning strategy questionnaire for middle school students, physical education classroom metacognitive process questionnaire
Interactive engagement	I discussed with classmates or the teacher how to practice more effectively.	Classroom observations
	I corrected my movements based on feedback.	Classroom observations
	I designed new tactics with my classmates.	Interviews, scale for the achievement of educational objectives in movement skills teaching
	I created new movements or movement combinations with my classmates.	Interviews, scale for the achievement of educational objectives in movement skills teaching, physical education classroom metacognitive process questionnaire

After constructing the item pool, the researchers first determined the time frame of the scale according to DeVellis's recommendations. Based on the purpose and needs of this study, the Cognitive Engagement in Physical Education Scale was designed to measure students' cognitive engagement in physical education classes. Therefore, the qualifier “during physical education class” was used in the scale. The researchers then determined the response format of the scale. Because the study aimed to measure the extent to which participants believed that each item accurately described their learning behaviors, a Likert-type scale was selected. The researchers also considered self-report measures to have higher validity than observation and rating scales when assessing cognitive engagement (Appleton et al., 2006; Greene, 2015). Considering scientific rigor, discrimination, convenience, and scope of use, a five-point Likert scale was used to score students' level of agreement.

After the initial item pool was generated, five experts in physical education were invited to review the items. The expert panel included senior professors, municipal teaching researchers, and experienced physical education teachers. The experts evaluated the items in terms of theoretical relevance, clarity of wording, developmental appropriateness for middle school students, consistency with the ICAP framework, and contextual suitability for physical education classes. After the expert review, three doctoral students in physical education and training were invited to further proofread and revise the initial items. The researchers then selected one class from a middle school in Jinan to conduct cognitive testing of the scale items. This testing was used to examine how participants understood, judged, and responded to the items. Based on their feedback, items with ambiguous wording, overlapping meanings, or insufficient relevance to physical education learning were revised or deleted. For example, in response to expert suggestions, the item “I set learning goals that are suitable for my own situation” was revised to “I set my own learning goals based on the teacher's learning goals and my own situation.” Based on students' feedback, the item “I repeatedly practice movements but do not think about how to improve them” was deleted because its wording was not sufficiently clear for middle school students. After the expert review, doctoral student proofreading, and cognitive testing, the initial 60-item pool was reduced to 48 items for the first survey administration.

The 48 items were organized according to the four ICAP modes: passive, active, constructive, and interactive engagement. After EFA and CFA, the factors were renamed based on their retained item content to better reflect the physical education context.

## 8 Steps 5 and 6: consider inclusion of validation items and administer items to a development sample

The purpose of adding validation items was to detect possible item defects and to examine the construct validity of the scale. For example, in self-report measures such as scales, social desirability is one of the most common sources of bias (Van de Mortel, 2008). When completing the questionnaire, students may give inaccurate responses because of teacher pressure, peer pressure, or social pressure. Item 9 was included as a reverse-coded item in the initial questionnaire. Its purpose was to reduce potential social desirability bias and to provide an additional check for response quality. During the exploratory analysis, however, this item did not align with the theoretically expected dimension and failed to fit the final factor structure. Therefore, it was removed from the final scale. Nevertheless, its inclusion was useful in the initial survey because it helped identify possible inattentive, patterned, or socially desirable responses and strengthened the data screening process.

In addition, previous studies have confirmed the relationship between self-efficacy and cognitive engagement (Walker et al., 2006). Therefore, physical education learning self-efficacy, adapted from Pintrich's 1990 Motivated Strategies for Learning Questionnaire (MSLQ), was added to the initial questionnaire as a validation measure. This was done to examine construct validity by testing the correlation between cognitive engagement in physical education and physical education learning self-efficacy.

The purpose of developing the Cognitive Engagement in Physical Education Scale was to examine middle school students' learning states in different types of physical education classes. Therefore, the selected participants needed to represent a relatively broad student population. Purposive and cluster sampling was used to recruit 907 students from 21 physical education classes across three grades in two middle schools in Jinan. The teaching content of these 21 physical education and health classes included basketball, football, volleyball, and other sports. A total of 907 questionnaires were distributed, and all 907 were returned, yielding a response rate of 100%.

Questionnaires were excluded if they showed continuous identical responses, all extreme responses, incomplete responses, or regular response patterns. After these exclusions, 855 valid questionnaires remained, with an effective response rate of 94.2%. To ensure data accuracy as much as possible, the data were entered using data entry software and a double-entry procedure by two researchers. Because this study collected data in two independent waves, the samples were used according to the purpose of each wave. The 341 valid questionnaires obtained in the first wave were used for item analysis and exploratory factor analysis. After item screening and preliminary structure identification, the 514 valid questionnaires obtained in the second wave were used for confirmatory factor analysis and reliability and validity testing. To strengthen the validity and generalizability of the findings, independent samples were designated for each stage of analysis, including EFA and CFA. This approach reduced potential biases from overlapping samples and enhanced the reliability of the scale's validation across different datasets.

## 9 Steps 7 and 8: evaluate the items and optimize scale length

Item evaluation is second only to item pool construction in importance during the scale development process. To evaluate our items, we conducted factor extraction methods and internal reliability testing in line with recommendations by DeVellis (2017).

Before conducting exploratory factor analysis, item analysis was first performed on the initial scale using Sample 1 (*N* = 341). The item analysis included extreme group comparisons, item-total correlations, and homogeneity tests. The evaluation indicators included critical ratios, item-total correlations, corrected item-total correlations, changes in Cronbach's alpha if an item was deleted, communalities, and factor loadings. Items 5, 10, 37, and 43 failed to meet the required criteria and were removed after item analysis (see Table 2).

**Table 2:** Item analysis results (*N* = 341).

Item	Extreme group comparison	Item-total correlation		Homogeneity test			Number of unmet criteria	Remarks
	Critical ratio	Item-total correlation	Corrected item-total correlation	Cronbach's alpha if item deleted	Communality	Factor loading		
A5	−3.964	**#0.373** ^**^	**#0.356**	0.953	**#0.162**	**#0.402**	**4**	**Recommended for deletion**
B10	−5.185	**#0.315** ^**^	**#0.279**	**#0.954**	**#0.083**	**#0.288**	**5**	**Recommended for deletion**
C37	−4.872	**#0.331** ^**^	**#0.304**	0.953	**#0.107**	**#0.328**	**4**	**Recommended for deletion**
Evaluation criteria	≥3.000	≥0.400	≥0.400	≤ 0.953	≥0.200	≥0.450		

0.953 is the internal consistency coefficient of the Cognitive Engagement in Physical Education Scale. The symbol # indicates values that did not meet the recommended evaluation criteria. ^**^ indicates *p* < 0.01.

After item analysis and comprehensive evaluation, these items were removed from the scale. The remaining items showed good discrimination. After item analysis, exploratory factor analysis was conducted to further examine the construct validity of the scale. Principal axis factoring with direct oblimin rotation was performed on the remaining 44 items. The initial analysis showed that the KMO value of the scale was 0.939, which was above the recommended threshold of 0.80. This indicated that the variables shared sufficient common factors. Bartlett's test of sphericity was significant, χ^2^ = 7502.127, df = 946, *p* < 0.001. These results indicated that the questionnaire was suitable for exploratory factor analysis.

Items were removed if they met one or more of the following criteria: factor loading below 0.40, cross-loading above 0.30, difference between primary and secondary loadings below 0.20, low communality, or theoretical inconsistency with the factor. Based on the above factor retention criteria, 30 items, including Items 3, 11, and 12, were removed sequentially. Exploratory factor analysis was then conducted again on the remaining 14 items. At this stage, the KMO value of the scale was 0.884. Bartlett's test of sphericity was significant, χ^2^ = 3,941.565, df = 91, *p* < 0.001. Four factors were extracted. The four-factor solution accounted for 54.344% of the variance. As shown in Table 3, all retained items loaded clearly on their corresponding factors in the pattern matrix, with factor loadings ranging from 0.505 to 0.895. These results supported the four-factor structure of the CEPE (see Table 3).

**Table 3:** Results of exploratory factor analysis (*N* = 341).

Factor	Item	Factor loading	Cronbach's alpha
Observation and execution	A1	0.820	0.783
	A2	0.881	
	A4	0.601	
	A6	0.621	
Cognitive processing	B18	0.611	0.701
	B19	0.572	
	B20	0.609	
Self-regulation	C33	0.643	0.777
	C34	0.809	
	C35	0.706	
	C36	0.505	
Collaborative innovation	D45	0.818	0.718
	D46	0.895	
	D47	0.666	

Factor loadings are based on the pattern matrix from principal axis factoring with direct oblimin rotation. The signs of the factor loadings for Collaborative Innovation were reversed for ease of interpretation. The overall Cronbach's alpha of the scale was 0.884 in the EFA sample.

After the specific dimensions and items of the Cognitive Engagement in Physical Education Scale were determined, the dimensions were renamed according to the content of the retained items. The original labels were revised as follows: passive engagement was renamed observation and execution, active engagement was renamed cognitive processing, constructive engagement was renamed self-regulation, and interactive engagement was renamed collaborative innovation. These revised names were used to better reflect the characteristics of teaching and learning in physical education classes.

**Figure 1:**
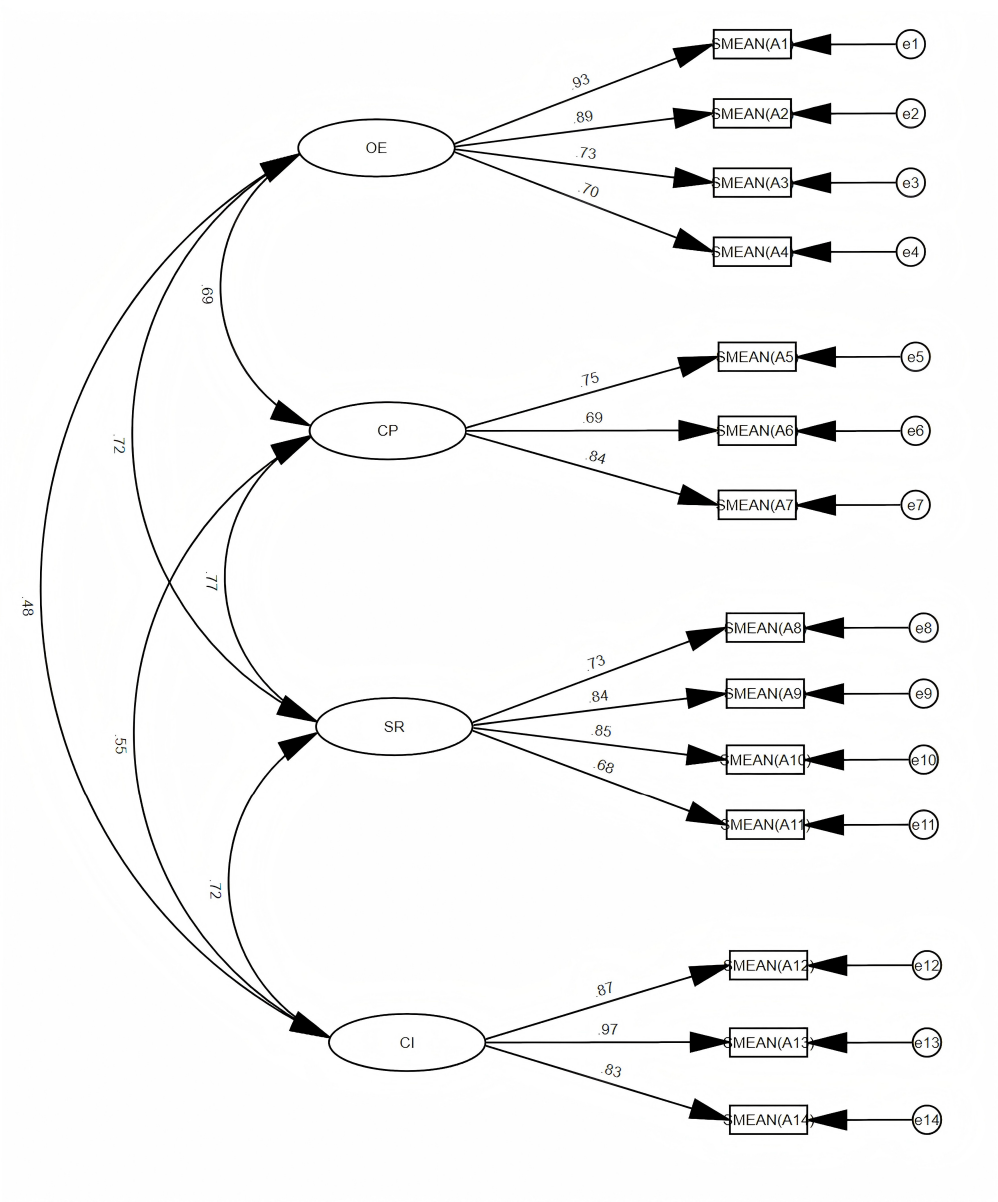
Structural equation modeling of the student cognitive engagement in physical education measurement model. OE = observation and execution; CP = cognitive processing; SR = self-regulation; CI = collaborative innovation.

After exploratory factor analysis, confirmatory factor analysis was conducted using Sample 2 (*N* = 514) to examine the structural validity of the scale. In the confirmatory factor analysis stage, maximum likelihood estimation was used to test the Cognitive Engagement in Physical Education Scale. The results showed that the model fit indices were acceptable: χ^2^/df = 4.396, RMSEA = 0.080, GFI = 0.919, CFI = 0.952, TLI = 0.938, IFI = 0.952, and SRMR = 0.041. The standardized measurement model is presented in Figure 1. These results indicated an acceptable model fit and supported the structural validity of the scale.

The standardized factor loadings of all items ranged from 0.68 to 0.97. All loadings were significantly higher than the minimum threshold of.40 (Watkins, 2018). The convergent validity of the scale was examined using average variance extracted. The AVE values of observation and execution, cognitive processing, self-regulation, and collaborative innovation were all above 0.50. The CR values of these four dimensions were all above 0.80. However, the square root of AVE for cognitive processing was 0.76, which was slightly lower than its correlation with self-regulation (0.77; see Table 4).

**Table 4:** Confirmatory factor analysis results (Part 1) (*N* = 514).

Factor name	CFA	CR	AVE	Observation and execution	Cognitive processing	Self-regulation	Collaborative innovation
Observation and execution	0.925	0.89	0.67	[0.82]			
	0.894						
	0.726						
	0.702						
Cognitive processing	0.745	0.81	0.58	0.69	[0.76]		
	0.690						
	0.844						
Self-regulation	0.727	0.86	0.61	0.72	0.77	[0.78]	
	0.841						
	0.854						
	0.681						
Collaborative innovation	0.874	0.92	0.80	0.48	0.75	0.72	[0.89]
	0.973						
	0.833						

Values in brackets represent the square root of AVE for each latent variable. All correlation coefficients were significant at the 0.01 level.

Because the Fornell–Larcker criterion indicated a marginal discriminant validity concern between Cognitive Processing and Self-Regulation, an additional competing model was tested in which these two factors were combined into a single factor. The merged model showed poorer fit than the proposed four-factor model, χ^2^/df = 6.39, RMSEA =0.103, CFI =0.921, TLI =0.902. The chi-square difference test also supported the superiority of the four-factor model, Δχ^2^ = 160.96, Δdf = 3, *p* < 0.01. These results suggested that, although Cognitive Processing and Self-Regulation were highly correlated, treating them as two separate constructs provided a better representation of the data. The results showed that the four-factor model had the best model fit (see Table 5).

**Table 5:** Confirmatory Factor Analysis Results (Part 2) (N = 514).

No.	Model	*χ^2^*	df	*χ^2^*/df	RMSEA	GFI	CFI	AGFI	IFI	TLI	NFI	Model comparison	Δ*χ^2^*	ΔDF
1	Four-factor model	312.124	71	4.396	0.080	0.919	0.952	0.881	0.952	0.938	0.939			
2	Three-factor model 1	567.970	74	7.675	0.102	0.853	0.902	0.791	0.902	0.879	0.889	2 VS. 1	255.846^***^	3
3	Three-factor model 2	817.491	74	11.047	0.140	0.768	0.852	0.671	0.852	0.818	0.840	3 VS. 1	505.367^***^	3
4	Three-factor model 3	473.084	74	6.393	0.103	0.879	0.921	0.829	0.921	0.902	0.907	4 VS. 1	160.96^***^	3
5	One-factor model	1,630.347	77	21.173	0.198	0.649	0.691	0.552	0.691	0.634	0.681	5 VS. 1	1,318.223^***^	6
6	Two-factor model	1,062.702	76	13.983	0.159	0.727	0.803	0.622	0.804	0.765	0.792	6 VS. 1	750.578^***^	5

Three-factor model 1 included OE + CP, SR, and CI. Three-factor model 2 included OE, CP, and SR + CI. Three-factor model 3 included OE, CP + SR, CI. The two-factor model included OE + CP and SR + CI. The one-factor model included OE + CP + SR + CI. ^***^ indicates *p* < 0.001. OE = Observation and execution, CP = Cognitive processing, SR = Self-regulation, CI = Collaborative innovation.

In addition, this study used the heterotrait-monotrait ratio (HTMT) to assess discriminant validity among the first-order constructs. HTMT below 0.90 was considered evidence of discriminant validity (Sharif-Nia et al., 2023). All HTMT values were below 0.90, providing additional evidence for discriminant validity among the four constructs (see Table 6).

**Table 6:** Heterotrait–monotrait ratio of discriminant validity evidence.

Construct	Collaborative innovation	Cognitive processing	Observation and execution	Self-regulation
Collaborative innovation				
Cognitive processing	0.552			
Observation and execution	0.475	0.745		
Self-regulation	0.764	0.775	0.765	

Furthermore, the retained items of Cognitive Processing and Self-Regulation were re-examined to determine whether there was substantial semantic overlap between the two dimensions. The items in Cognitive Processing focused on mental rehearsal, movement imitation, and tactical simulation, whereas the items in Self-Regulation focused on goal setting, strategy adjustment, learning monitoring, and reflection on practice errors. Therefore, no retained item was considered to show substantial semantic overlap requiring deletion.

Although the Fornell–Larcker criterion was not fully satisfied for the cognitive processing and self-regulation factors, the difference was marginal. Additional evidence from HTMT values, competing model comparisons, and item-content examination supported the discriminant validity of the four-factor structure. Therefore, the discriminant validity of the scale was considered acceptable, although future studies should further examine the distinction between cognitive processing and self-regulation.

In addition, Pearson correlation analysis showed that the CEPE total score and subscale scores were positively correlated with physical education learning self-efficacy (see Table 7). These positive correlations provided evidence of criterion-related validity.

**Table 7:** Correlation between the CEPE and physical education learning self-efficacy.

Variable	Cognitive engagement in physical education	Observation and execution	Cognitive processing	Self-regulation	Collaborative innovation	Physical education self-efficacy
Cognitive engagement in physical education	1					
Observation and execution	0.794^**^	1				
Cognitive processing	0.799^**^	0.625^**^	1			
Self-regulation	0.905^**^	0.664^**^	0.638^**^	1		
Collaborative innovation	0.814^**^	0.432^**^	0.471^**^	0.675^**^	1	
Physical education self-efficacy	0.498^**^	0.348^**^	0.372^**^	0.457^**^	0.453^**^	1

^**^ Correlation is significant at the 0.01 level (two-tailed).

## 10 Discussion

As a key indicator of students' internal mental activity and depth of knowledge construction in physical education learning, cognitive engagement in physical education can reflect the quality of physical education teaching and student learning. Measuring students' cognitive engagement in physical education in a simple and accurate way is an important basis for designing effective teaching strategies, improving students' cognitive engagement, and enhancing the quality of physical education teaching (Li, 2021). However, existing instruments for measuring cognitive engagement in physical education still have limitations in terms of contextual specificity, measurement accuracy, and practical applicability.

In this study, the researchers developed an ICAP-based scale to measure students' cognitive engagement in physical education. This scale helps address the limitations of existing measurement tools for cognitive engagement in physical education. The Cognitive Engagement in Physical Education Scale developed in this study includes four dimensions: observation and execution, cognitive processing, self-regulation, and collaborative innovation. This structure is consistent with the ICAP framework and previous research on cognitive engagement. The reliability and validity tests indicated that the scale showed acceptable psychometric properties. First, item analysis, including critical ratios, item-total correlations, and factor loadings, showed that the scale consisted of distinct and internally consistent items. Second, exploratory factor analysis showed that the scale had good overall internal consistency, with a Cronbach's alpha of 0.884. The final scale included four subscales and 14 items. Third, confirmatory factor analysis supported the structural validity of the scale. The model fit indices, including χ^2^/df, CFI, TLI, and RMSEA, met the recommended criteria, which indicated that the model fit was acceptable and supported the structural validity of the scale. Finally, convergent and discriminant validity were examined using the Fornell-Larcker criterion (Fornell and Larcker, 1981). The AVE values for observation and execution, cognitive processing, self-regulation, and collaborative innovation were all above the critical value of 0.50. The CR values for these four dimensions were also above the critical value of 0.70, indicating good convergent validity. In addition, the square roots of AVE for observation and execution, self-regulation, and collaborative innovation were greater than their corresponding inter-factor correlations. However, the square root of AVE for cognitive processing was slightly lower than its correlation with self-regulation (0.76 vs.0.77). Given that the difference was only 0.01, and that the CR (0.81), and AVE (0.58) values for this dimension met the recommended criteria. In addition, HTMT values, competing model comparisons, and item-content examination provided additional evidence for discriminant validity. These results suggest that the discriminant validity of the scale was generally acceptable.

### 10.1 The framework of measurement

The Cognitive Engagement in Physical Education Scale developed in this study was based on the ICAP framework. The final 14-item scale is presented in Table 8. It divides students' cognitive engagement in physical education into four dimensions: observation and execution, cognitive processing, self-regulation, and collaborative innovation. These four dimensions are not simply parallel to one another. Instead, they are interrelated and progressive. They reflect a process in which students move from lower-level to higher-level engagement and from observable behavioral participation to deeper cognitive construction in physical education learning (Han et al., 2022).

**Table 8:** Revised cognitive engagement in physical education scale for middle school students.

Dimension	Item
Observation and execution	I watched the teacher's movement demonstration throughout the class.
	I listened to the teacher's explanation of the key points of the movement.
	I repeatedly practiced the movement as required.
	I followed the teacher's instructions in class.
Cognitive processing	When the teacher explained and demonstrated a movement, I tried to mentally perform the movement.
	When the teacher explained and demonstrated a movement, I imitated it on my own.
	When the teacher explained a tactic, I tried to mentally perform the tactic.
Self-regulation	I set my own learning goals based on the teacher's goals and my own situation.
	I adjusted my learning strategies in a timely manner.
	I monitored my own learning.
	I reflected on the mistakes I made during practice.
Collaborative innovation	I designed new practice methods with my classmates.
	I designed new tactics with my classmates.
	I created new movements or movement combinations with my classmates.

The observation and execution dimension mainly includes paying attention to the teacher's movement demonstrations, following classroom instructions, and listening to explanations of movement techniques. Observation and execution represent the most basic form of cognitive engagement in physical education learning. Physical practice is one of the key features of physical education learning. In physical education, students first need to obtain movement-related information through observation, listening, and imitation (Wulf et al., 2010). At this stage, students' observation and execution of classroom instructions are not merely physical actions. They also involve cognitive processes such as attention allocation, movement information reception, and movement memory.

The cognitive processing dimension mainly includes motor imagery, tactical simulation, and movement imitation. Compared with observation and execution, cognitive processing represents a higher and deeper level of engagement. It reflects students' internal processing and reconstruction of movement-related information. After receiving information from the teacher's movement demonstrations, students process this information through internal mental activities such as motor imagery. This helps them better understand and master motor skills. The cognitive processing dimension further shows that students' thinking activities in physical education are closely linked to their actions, perceptions, and motor representations (Munzert et al., 2009). This reflects the embodied nature of cognitive engagement in physical education.

The self-regulation dimension mainly includes setting learning goals based on teaching goals and personal conditions, monitoring learning, and reflecting on practice errors. At this stage, students no longer only receive teacher guidance passively. Instead, they actively plan, monitor, and reflect on their own physical education learning. It is worth noting that self-regulation in physical education is highly immediate. For example, when practicing movements such as the three-step layup or standing long jump, students need to adjust their movements or learning strategies immediately based on their current performance (Cleary and Zimmerman, 2001).

It is particularly important to clarify the distinction between cognitive processing and self-regulation. Cognitive processing mainly reflects students' internal processing of information related to technical movements and game tactics. It mainly involves mental rehearsal, tactical simulation, and motor representation. These cognitive processing activities can help students understand and master physical education and health knowledge as well as motor skills. In contrast, self-regulation mainly reflects students' metacognitive control over their own physical education learning process. It mainly involves reflection, monitoring, goal setting, and learning strategies. Therefore, cognitive processing answers the question of how students mentally process information related to movements and tactics, whereas self-regulation answers the question of how students control and improve their own learning process.

In the field of physical education, the high correlation between cognitive processing and self-regulation is theoretically understandable. Unlike academic subject learning, physical education learning is immediate and synchronous. Students often need to process movement information while monitoring and adjusting their learning performance. For example, when learning motor skills, students may mentally simulate a movement while comparing their own performance with the teacher's demonstration and adjusting their movement accordingly. Therefore, in actual physical education learning, these two dimensions are closely connected. However, they still differ fundamentally. Cognitive processing reflects the information-processing function of cognitive engagement, whereas self-regulation reflects the metacognitive regulatory function of cognitive engagement.

The collaborative innovation dimension mainly includes designing new practice methods, designing new tactics, and creating new movements with classmates. This stage represents the highest level of cognitive engagement in physical education learning. At this stage, students are no longer satisfied with processing existing knowledge. Instead, they build on existing knowledge and co-construct new knowledge through interaction and negotiation with classmates. Physical education learning is highly situated. The acquisition, generation, and development of physical education knowledge often occur through group practice, peer guidance, and tactical cooperation in games (Zach et al., 2023; Schulze and Von Huth, 2022). The emergence of the collaborative innovation dimension is highly consistent with interactive engagement in the ICAP framework. It also shows that cognitive engagement in physical education is not only an individual mental process but also has clear social and interactive features (Whipp et al., 2015).

### 10.2 Theoretical implications

Compared with similar studies, this study further extends the application of the ICAP framework to physical education. It demonstrates the adaptability of the ICAP framework across different subject areas. It also provides a basis for future research to further examine the theoretical value of the ICAP framework in physical education. In addition, this study offers a new perspective for research on cognitive engagement in physical education. In terms of measurement and theory, the findings support the view that cognitive engagement in physical education is a multidimensional construct (Fredricks et al., 2004; Greene, 2015; Li, 2021). Therefore, the analysis, measurement, and intervention of cognitive engagement in physical education should be conducted from multiple perspectives. Any measurement or intervention based on a single dimension may be incomplete. In terms of scale development, the CEPE is a new tool for measuring students' cognitive effort during physical education learning. It fills a gap in local research. Compared with other measurement tools, the CEPE developed in this study has at least three advantages. First, driven by theory and supported by empirical data, it classifies students' cognitive engagement in physical education into four levels: observation and execution, cognitive processing, self-regulation, and collaborative innovation. This allows students' levels of cognitive engagement in physical education to be distinguished more accurately. Second, the scale items were mainly derived from established measurement tools and were refined through classroom observations. Therefore, the item content can closely reflect students' learning behaviors in physical education and measure their cognitive engagement more accurately. Third, the scale focuses on students' behavioral performance in physical education classes. Compared with tools that focus mainly on students' internal psychological states, this approach may reduce measurement error caused by social desirability to some extent. As a result, it may further improve measurement accuracy.

### 10.3 Practical implications

Relevant theory and empirical evidence show that students' cognitive engagement in physical education is an important factor influencing their learning outcomes. Low levels of cognitive engagement may be one reason for poor teaching effectiveness in physical education and health courses (Wang and Zhu, 2022). However, due to the lack of convenient and context-specific measurement tools, cognitive engagement has often been insufficiently considered in instructional evaluation and intervention.

The Cognitive Engagement in Physical Education Scale developed in this study showed acceptable reliability and validity in the present sample. It may provide a useful tool for assessing students' cognitive engagement in physical education and for evaluating instructional interventions in similar physical education contexts. By examining students' scores on different dimensions, researchers and physical education teachers may better understand whether students' engagement is mainly reflected in basic observation and execution, internal cognitive processing, self-regulation, or collaborative innovation. This dimensional information can help teachers identify possible weaknesses in students' learning processes and design more targeted instructional activities. In practice, PE teachers can use dimension-level CEPE scores to identify whether a class or a group of students shows relatively low engagement in specific areas. Teachers may then adjust instructional strategies according to the weaker dimensions and use repeated assessment to examine whether students' cognitive engagement improves after instructional changes.

For example, students with low scores on Observation and Execution may need clearer demonstrations, more explicit task instructions, or additional opportunities for guided practice. Students with low scores on Cognitive Processing may benefit from prompts that encourage motor imagery, tactical thinking, or attention to key movement details. Students with low scores on Self-Regulation may need support in setting learning goals, monitoring their performance, adjusting learning strategies, and reflecting on practice errors. Students with low scores on Collaborative Innovation may benefit from inquiry-based teaching, group-based practice, peer discussion, cooperative problem-solving, or small-sided game tasks that encourage communication and tactical co-construction.

However, these practical applications should be interpreted cautiously. The CEPE has so far been validated only with middle school students from two schools in Jinan, China. Therefore, further validation across different regions, school types, grade levels, and physical education contexts is needed before the scale is used for broad educational evaluation or policy-level decision-making. With further validation in more diverse school contexts, the scale may provide useful information for understanding classroom learning quality and supporting the improvement of physical education teaching.

## 11 Limitations and future research directions

Several limitations should be noted. First, this study represents an initial development and validation of the CEPE. Although the scale showed acceptable reliability and validity, the distinction between Cognitive Processing and Self-Regulation should be further examined because these two dimensions were highly correlated.

Second, the sample was drawn from two middle schools in Jinan with relatively strong physical education programs, which may limit the generalizability of the findings and introduce selection bias. Future studies should validate the CEPE in more diverse school contexts, including average or lower-performing schools, rural and urban schools, and less structured physical education environments.

Third, measurement invariance across gender, grade level, and different physical education contexts was not examined because the subgroup structure was not sufficiently balanced for stable multi-group CFA. Future studies should recruit larger and more balanced samples to test whether the CEPE functions equivalently across different student groups and instructional contexts.

Fourth, although experts reviewed the initial item pool, formal quantitative content validity indices were not calculated. Future studies should use structured expert rating procedures to provide stronger content validity evidence.

Finally, the CEPE was developed and validated among Chinese middle school students using self-reported data. Future research should combine self-report with other methods, such as classroom observation or teacher ratings, and examine the scale's cross-cultural applicability.

## 12 Conclusion

This study extended the application of the ICAP framework to physical education and developed an ICAP-based tool for measuring students' cognitive engagement in PE. The results showed that the CEPE consisted of four dimensions and 14 items and demonstrated acceptable reliability and validity in the present sample. The CEPE may help researchers and PE teachers assess students' cognitive engagement across different dimensions, including observation and execution, cognitive processing, self-regulation, and collaborative innovation. However, further validation in more diverse school contexts and student groups is needed before the scale is used for broader educational evaluation.

## Data Availability

The raw data supporting the conclusions of this article will be made available by the authors, without undue reservation.
